# Research progress in delineating the pathological mechanisms of *GJB2*-related hearing loss

**DOI:** 10.3389/fncel.2023.1208406

**Published:** 2023-06-02

**Authors:** Yujun Wang, Yuan Jin, Qiong Zhang, Ying Xiong, Xiang Gu, Shan Zeng, Wei Chen

**Affiliations:** ^1^Department of Intensive Care Unit, The Central Hospital of Wuhan, Tongji Medical College, Huazhong University of Science and Technology, Wuhan, China; ^2^Department of Otorhinolaryngology–Head and Neck Surgery, The Central Hospital of Wuhan, Tongji Medical College, Huazhong University of Science and Technology, Wuhan, China

**Keywords:** *GJB2* gene, gap junction protein, hearing loss, K^+^ circulation, cochlear

## Abstract

Hearing loss is the most common congenital sensory impairment. Mutations or deficiencies of the *GJB2* gene are the most common genetic cause of congenital non-syndromic deafness. Pathological changes such as decreased potential in the cochlea, active cochlear amplification disorders, cochlear developmental disorders and macrophage activation have been observed in various *GJB2* transgenic mouse models. In the past, researchers generally believed that the pathological mechanisms underlying *GJB2*-related hearing loss comprised a K^+^ circulation defect and abnormal ATP-Ca^2+^ signals. However, recent studies have shown that K^+^ circulation is rarely associated with the pathological process of *GJB2*-related hearing loss, while cochlear developmental disorders and oxidative stress play an important, even critical, role in the occurrence of *GJB2*-related hearing loss. Nevertheless, these research has not been systematically summarized. In this review, we summarize the pathological mechanisms of *GJB2*-related hearing loss, including aspects of K^+^ circulation, developmental disorders of the organ of Corti, nutrition delivery, oxidative stress and ATP-Ca^2+^ signals. Clarifying the pathological mechanism of *GJB2*-related hearing loss can help develop new prevention and treatment strategies.

## Introduction

Changes in the genes encoding gap junction proteins (connexins, Cx) are the most common genetic causes of hearing loss (He et al., 2017; Chai et al., 2022). Thus far, 21 human gap junction proteins have been identified. These are transmembrane proteins consisting of six identical or different gap junction protein monomers that form a hemichannel, which called connexon; the two connexons then form the gap junction channel (GJCs) between adjacent cell. GJCs allow for material exchange between adjacent cells, and small molecules (<1.4 kDa) such as ions, adenosine triphosphate (ATP) and miRNA can pass through them. Cx26 encoded by the *GJB2* gene and Cx30 encoded by the *GJB6* gene, the most abundant gap junction proteins in the inner ear, are expressed in the support cells of the organ of Corti (OC), the basal and intermediate cells of the stria vascularis, and fibroblasts in the spiral ligament. GJCs junctions formed by Cx26 plays an important role in the development, formation and maintenance of auditory function, and material exchange in inner ear cells. Up to now, more than 100 mutations associated with human hearing loss have been identified in the region of *GJB2* gene. *GJB2* mutation has been observed to be involved in the pathogenesis of hereditary deafness, delayed deafness and presbycusis. Although many valuable pathological phenomena have been elucidated using *Gjb2* gene knockdown (KD) and *Gjb2* mutant transgenic mice, the main pathological mechanism of hearing loss caused by *GJB2* mutation is still unclear. This paper summarizes recent progress in research on *GJB2*-related hearing loss, attempting to identify the pathological mechanism of hearing loss caused by Cx26 mutation.

## K^+^ circulation

**FIGURE 1:**
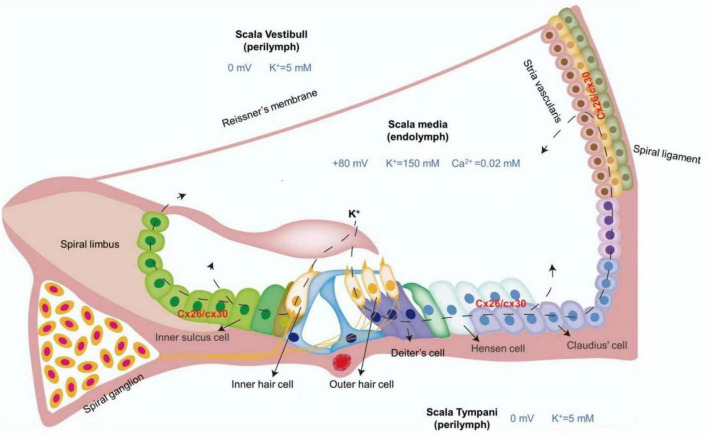
Diagram of K^+^ circulation.

The cochlea, a coiled structure located in the ventral region of the inner ear, acts as the primary structure for the perception of sound. The cochlea consists of three internal cavities filled with lymph fluid: from top to bottom, the scala vestibuli (SV), scala media (SM), and scala tympani (ST). The SV and ST contain perilymph fluid and traffic with each other through the cochlear aperture at the top of the cochlea (Jagger and Forge, 2015), while the SM contains endolymph fluid. Endolymph fluid and perilymph fluid constitute unique ionic environments (Zhang et al., 2023). Perilymph is similar to extracellular fluid, with a high concentration of Na^+^ and a low concentration of K^+^, and endolymph is similar to intracellular fluid, with a low concentration of Na^+^ and a high concentration of K^+^; this leads to a difference in potential between different parts of the cochlea in the resting state (Zhao, 2017). As endolymph contains up to 150 mM K^+^, 2 mM Na^+^, and 20 mM Ca^2+^, it exhibits a potential of approximately + 80 mV relative to the neighboring extracellular spaces. The cell bodies of hair cells are immersed in endolymph. When sound is transmitted to the inner ear, the mechanical vibration of the basilar membrane makes the hair cell bundle deflect and open the mechanically sensitive channel at the top of the stereocilia (Chai et al., 2018). Driven by endocochlear positive potential, K^+^ in the endolymph passes through the mechano-transduction channels in the hair cells’ hair bundles, thus generating auditory receptor current and potential. The K^+^ entering the hair cells passes through their basal lateral membrane via the K^+^ channel and is discharged. This K^+^ reaches the lateral cochlear wall through the GJCs network composed of Cx26 and Cx30 between Deiters cells and epithelial cells in the outer lymph or basement membrane and is finally discharged into the endolymph (Figure 1; Nin et al., 2012). Cx26 mutation is thought to damage the inner ear GJCs function and destroy the cochlear GJCs-mediated K^+^ circulation, resulting in toxic K^+^ accumulation around hair cells and eventually leading to hair cell degeneration and hearing loss. This process was once considered the main pathological mechanism of *GJB2*-related hearing loss. However, this model lacks sufficient experimental evidence and its theory is based on indirect evidence related to the expression of ion channels and connexins. This model also cannot explain why either mutated Cx26 or Cx30 cannot be compensated for by another kind of connexin; for example, Cx30 still exists in the cochlea of *Gjb2* KD mice and should be located in the same region. Additionally, GJCs in the inner ear of *Gjb2* gene KD mice retains permeability, which suggests that it theoretically still has K^+^ circulation ability. More importantly, hearing loss was detected in a *Gjb2* R75W mouse model, but its endolymphatic potential was still within the normal range, which showed that impaired K^+^ circulation was not the main pathological mechanism of deafness caused by *Gjb2* mutation but only a concomitant phenomenon (Inoshita et al., 2008). Moreover, an important aspect of the K^+^ circulation hypothesis is the degeneration of hair cells caused by the accumulation of K^+^ around them following Cx26 mutation. However, some studies have shown that the hearing loss caused by *Gjb2* mutation occurs before the degeneration of hair cells, which also indicates that the K^+^ circulation is not the pathogenic mechanism of *GJB2*-related hearing loss (Liang et al., 2012).

## Development of the organ of Corti

In independent lines of Cx26 null mice, researchers observed that the tunnel of Corti (TC) and Nuel’s space (NS) never opened and the postnatal development of OC was blocked (Minekawa et al., 2009). Morphological abnormalities in TC and NS were also observed in a *Gjb2* R75W transgenic mouse model (Zhang et al., 2005). Thus, in four different *Gjb2* transgenic mouse models, the postnatal development of OC was observed to stop before cell death in this organ. The opening of the TC and the formation of Nuel’s space are important events for the facilitation of hearing. The delayed or impaired development of normal physiological structures can cause serious hearing loss (Forrest et al., 2002). Dysplastic OC were observed in patients with keratitis-ichthyosis-deafness (KID) syndrome with a heterozygous *GJB2* G45E variant (Griffith et al., 2006). Additionally, unopened TC and abnormal NS were observed in both *Gjb2* variant carriers and mice. This cross-species phenomenon suggests that abnormalities in OC caused by the absence of Cx26 with normal physiological function may form the pathological basis for hearing defects.

The mechanism by which *GJB2* mutation causes malformation of OC has not been fully clarified. Current research suggests that the opening of TC is related to the development of inner and outer pillar cells. Inoshita et al. observed that pillar cell bodies were differentiated from the surrounding cells during the expansion of TC at postnatal day (P)8 in both non-transgenic mice and *Gjb2* R75W mice (Inoshita et al., 2008). However, there was a significant decrease in the number of microtubules in the inner column cells of the deformed OC in *Gjb2* R75W mutant mice, while abundant microtubules were formed parallelly in non-transgenic mice. Chen et al. observed pathological changes in the cochlea of transgenic mice through the conditional KD of cochlear Cx26 at P0 and P8 (Chen et al., 2018). In the P8 KD group, *Gjb2* KD did not significantly affect the morphology of OC. In the P0 KD group, TC and NS did not form during P5–P7 and the phalangeal process of Deiters cells did not develop into finger-like structures. The formation of microtubules in the pillar cells was significantly reduced, as was the amount of acetylated α-tubulin in these cells. These results suggest that the KD of the *Gjb2* gene during the early postnatal period in mice may cause disorders in the cytoskeletal development of pillar cells. Liu X. et al. (2021) also observed that the KD of the *Gjb2* gene during the early postnatal period in mice resulted in the reduction of F-actin levels in pillar cells. These studies suggest that the malformation of OC caused by Cx26 KD may be related to the disordered development of the cytoskeleton of pillar cells. Qiu et al. (2022) showed that the KD of cochlear Cx26 led to the malformed assembly of non-centrosomal microtubule-organizing centers far from the centrosome, accompanied by a decrease in the microtubule arrays emitted by abnormal non-centrosomal microtubule-organizing centers. The malformation of non-central microtubule-organizing centers in Cx26-deleted mice may lead to problems in the capture and anchoring of the microtubules of column cells, resulting in the malformation of OC.

**FIGURE 2:**
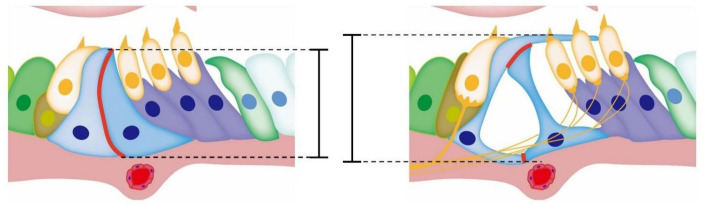
Diagram of the development of OC. The red mark denotes the adhesive connection between pillar cells. During the opening of TC, the adhesive junctions between pillar cell bodies gradually degrade and gaps appear in the originally tight cell membrane. With the development and growth of the cytoskeleton of inner and outer pillar cells, the height of OC increases, while TC appears between the inner and outer pillar cells.

The opening of TC may be related to the reduction of cell connections between the inner and outer pillar cells. The reduction of adhesion via E-cadherin between pillar cells is considered a key event (Whitlon, 1993; Johnen et al., 2012). Defourny et al. (2019) showed that Eph receptor A4 (EphA4) could bind with A Disintegrin And Metalloproteinase 10 (ADAM10) to promote the destruction of adhesion between inner and outer pillar cells based on E-cadherin. In the presence of defective EphA4 or an ADAM10 inhibitor, the adhesion junctions between pillar cells based on E-cadherin cannot be degraded normally. The cutting effect of the molecular scissors ADAM10 on cadherin was also found in other tissues and cells (Maretzky et al., 2005, 2008; Guerra et al., 2021). Zhang et al. (2022) observed that OC opened earlier in mice treated with triiodothyronine (T3) than in normal mice, accompanied by the formation of acetylated microtubules and a decrease of the adhesion junction molecule P-cadherin but not E-cadherin in pillar cells. This is consistent with the conclusions of other researchers. In general, the current research supports the notion that the development of the cytoskeleton of the pillar cells supports the height and deformation of the columnar cells, and the reduction of the adhesive connections formed by E-cadherin or P-cadherin between the columnar cells also participates in the opening of OC during its development and maturation (Figure 2). In addition, the abnormal development of the cytoskeleton may lead to other problems, such as affecting the transport of membrane proteins and thus affecting other cell functions. The *Gjb2* variant or *Gjb2* gene KD may affect the degradation of adhesion junctions between pillar cells through unknown pathways The Tspan protein family reported in other systems has a regulatory effect on ADAM10 splicing E-cadherin. Thus, further research on the relationship between Cx26 and Tspan may be valuable (Reyat et al., 2017; Seipold et al., 2018).

## Nutrient delivery

The spiral vessel exists at the bottom of the avascular sensor epiphyllum in the mammalian cochlea, but it is generally believed that blood vessel networks in the stria vascularis, spiral ligaments and spiral limbus also transport glucose and other metabolites to the sensory epithelium and sensory hair cells through GJCs between supporting cells (Santos-Sacchi and Dallos, 1983). Studies have shown that mice carrying *Gjb2* homozygous defects die in the womb during the short period from early to middle pregnancy (Gabriel et al., 1998). This is because the placenta of Cx26-deficient mouse embryos is severely impaired in its ability to absorb glucose and other nutrients from maternal blood, hence inhibiting rapid organogenesis during the second trimester of pregnancy. Intercellular coupling via supporting cells’ GJCs provides an activity-dependent intercellular pathway for the delivery of nutrients from blood vessels to distal neurons. Fetoni et al. (2018) assessed the glucose uptake of *Gjb2*^+/–^ mice with an insufficient Cx26 level using a fluorescent non-metabolizable D-glucose analyzer, 2-NBDG, and found that the 2-NBDG fluorescence emission in the stria vascularis of *Gjb2*^+/–^ mice was significantly reduced compared with *Gjb2*^+/–^ mice. Like Cx26, Cx30, which is most abundantly expressed in the cochlea and co-located with Cx26, has also been proven to play an indispensable role in the energy supply of the cochlear sensory epithelium. Nutritional deficiency affects ATP production and normal function and causes oxidative stress and other damage.

## Oxidative stress

Reactive oxygen species (ROS) play an important role in cell biology (He et al., 2017, 2020; Liu W. et al., 2021). ROS are indispensable in physiological processes such as protein phosphorylation, ion channels and the redox regulation of transcription factors (Zhong et al., 2020). However, prolonged exposure to high ROS concentrations may cause non-specific damage to proteins, lipids and nucleic acids, leading to the loss or destruction of cell function (Wang et al., 2022). Like the plasma membrane half-channel formed by Cx43 through which glutathione (GSH) is released by brain astrocytes to help neurons resist oxidation, the intercellular connection formed by supporting cells in the cochlea also has positive significance for GSH release and oxidation. Fetoni et al. (2018) compared the mRNA expression profile of *Gjb2*^–/–^ mice at P5 with age-matched *Gjb2* loxP/loxP mice. The results showed the activation of metabolic signaling pathways such as oxidative metabolism, the antioxidant defense system and glutathione metabolism (Fetoni et al., 2018). The amount of GSH released from cochlear explants from P5 *Gjb2*^–/–^ mice was significantly lower than that from *Gjb2* loxP/loxP mice. The total amount of glutathionylated proteins in the cochlea and their expression in the spiral ganglion neurons (SGNs), OC and stria vascularis were significantly reduced in 6-month-old *Gjb2*^+/–^ mice compared with *Gjb2* loxP/loxP mice. These findings indicate that the function of the cochlear antioxidant defense system in Cx26-deficient mice is impaired. Dihydroethidium (DHE, a lipophilic cell-permeable dye that is rapidly oxidized to ethidium in the presence of superoxide free radicals) was used to compare ROS content in the cochlea of *Gjb2*^–/–^ mice at P5 and age-matched *Gjb2* loxP/loxP mice. The results showed that the DHE signal in the cochlea of Cx26-deficient mice was significantly higher. Similarly, in the cochlea of *Gjb2*^+/–^ mice aged 2, 6, and 12 months, the staining signals of DHE and 4-hydroxy-2-nonenal (4-HNE, a lipid peroxidation product) were also observed to be significantly higher than those in age-matched *Gjb2* loxP/loxP mice, while the 2-month-old *Gjb2*^+/–^ mice showed no significant hearing loss compared with *Gjb2* loxP/loxP mice. Moreover, the antioxidant stress signaling pathway Nrf2/ARE and its response product, the HO-1 enzyme, are also activated in the cochlea of *Gjb2*^+/–^ mice. These findings indicate that oxidative metabolism and ROS play an important role in hearing loss caused by Cx26 KD. A significantly higher ROS level was also observed in the cochlea of Cx30 null mice (Teubner et al., 2003). A genome-wide association studies (GWAS) carried out in a large cohort of 4,091 individuals originating from Europe, Caucasus and Central Asia, with hearing phenotype (including 1,076 presbycusis patients and 1,290 healthy matched controls), showed a statistically significant association with two genes (PRKCE and TGFB1) related to the Nrf2 pathway, further supporting the hypothesis that elements of the Nrf2 pathway are essential for hearing maintenance (Fetoni et al., 2018).

## ATP release and Ca^2+^ signals

ATP release and the associated calcium signal transduction play an important role in inner ear development and hearing (Bobbin and Thompson, 1978; Kujawa et al., 1994; Muñoz et al., 1995; Housley et al., 1999). ATP can be used as a neurotransmitter, and its receptors can be divided into two major classes of purinergic receptors (P1 receptors and P2 receptors), each with multiple subtypes (Huang et al., 2010). P2 receptors are classified into two major groups, P2X and P2Y. P2X receptors are non-selective ATP-gated ion channels with high permeability for Na^+^, K^+^, and Ca2^+^, while P2Y receptors are G protein-coupled metabotropic receptors that activate phospholipase C (PLC), resulting in the activation of the second messengers diacylglycerol and inositol trisphosphate (IP3). The P2Y1, P2Y2, P2Y4, P2Y6, and P2Y12 receptors have been reported to be expressed in the sensory and non-sensory cells of OC and the spiral ganglion neurons of developing rat cochlea (Huang et al., 2010), while P2X2, P2Y4, and P2X7 are distributed on the OHC surface and correlated with the electrical activity of the OHC (Nikolic et al., 2003; Tritsch et al., 2007, 2010; Dayaratne et al., 2014). When the purinergic receptor P2Y of inner ear cells is activated by ATP, PLC-dependent IP3 is released and activates the endoplasmic reticulum IP3 receptor, which promotes the release of endoplasmic reticulum Ca^2+^ and raises the cytosolic free Ca^2+^ concentration, thus inducing Ca^2+^ signal transduction. ATP has been observed to trigger Ca^2+^ waves in the Kolliker’s organ of the immature OC as well as Ca^2+^ APs in IHCs (Tritsch et al., 2007, 2010; Anselmi et al., 2008; Majumder et al., 2010). ATP-induced IP3-dependent intercellular Ca^2+^ signals propagate radially across this cochlear cellular network at a uniform speed of 10 to 15 μm/s (Gale et al., 2004; Piazza et al., 2007). In the developing cochlea, the waves of ATP released from non-sensory cells of the greater epithelial ridge cause Ca^2+^ action potentials of immature inner hair cells to occur in brief bursts during episodes of spontaneous depolarization, which promotes the maturation of hair cells and SGNs and refines axonal projections from the cochlea to the central nervous system (Tritsch et al., 2010). In addition, exogenous ATP can depolarize type II neurons both directly and by evoking glutamate synaptic inputs from external hair cells, but the effect of this type of ATP decreases with age at birth (Greenwood et al., 2007).

Stout et al. (2002) reported that the level of extracellular ATP in a culture of Cx43-transfected cells was higher than that in a culture of the parent cells, which suggested that ATP release in astrocytes and glioma cells might be mediated by the Cx43 half-channel. Zhao and Santos-Sacchi (1999) showed that connexin hemichannels in cochlear supporting cells could release ATP under physiological conditions, with the level corresponding with physiological ATP concentrations in the cochlea, thus accounting for the submicromolar concentrations measured in cochlear fluids *in vivo* (Inoshita et al., 2008). Extracellular ATP could alter OHC electromotility through the activation of P2 purinergic receptors. Therefore, Cx26 dysfunction affects OHC electromotility and hearing function. A cochlear organotypic culture study showed that connexins make up the channels responsible for ATP-induced Ca^2+^ waves in the outer sulcus over Panx1 and P2X7 (Zhao and Santos-Sacchi, 1999). Additionally, the infusion of ATP into the cochlea can reduce and inhibit the otoacoustic emission of cochlear microsounds, complex action potential and distortion products, which can reduce active cochlear mechanics and hearing sensitivity. These studies suggest that GJc plays an important role in the ATP release and intercellular Ca^2+^ signaling transmission of inner ear Sertoli cells. In addition, a decrease in Ca^2+^ concentration increases the opening of chemical channels, which promotes ATP release from the cytoplasm to the endolymph through connexin hemichannels. Mechanical stimulation also induces hemichannels to release ATP. As sound-induced mechanical vibration increases, hemichannel ATP release increases to reduce OHC electromotility as a possible protective mechanism, which can enhance auditory sensitivity when the sound intensity is low (Brownell et al., 1985; Ashmore, 1987; Dallos, 1992; Anselmi et al., 2008; Tritsch et al., 2010).

The study of Ca^2+^ signals and ATP release requires drugs such as Cx channel blockers and Ca^2+^ indicators. The pharmacological compounds used in most studies involving connexin are not specific and have been proven to affect the activities of various other channels, while the putative conduits expressing ATP in inner ear cells may include connexin channels, P2X7 receptors (P2X7Rs), pannexin channels, anime channels, vessels and transports (Spray et al., 2006). Thus, rigorous standards must be applied for the detection of hemichannel expression and function.

## Summary and outlook

Gap junction channels composed of Cx26 allow for the exchange of small molecules such as glucose, second messengers, ions and miRNA. Previous studies have suggested that Cx26 plays a role in the K^+^ circulation of the inner ear, maintaining the high potential of the inner ear and the electrical activity of hair cells. Mutant Cx26 is unable to support K^+^ circulation, thus impairing the amplification function of the cochlea. However, recent studies have shown that K^+^ circulation disorder is not the main pathological mechanism of Cx26 mutation-related deafness. Researchers believe that Cx26 mutation causes changes in Ca^2+^ signaling and ATP release as well as columnar cell cytoskeletal developmental disorders, all of which contribute to the occurrence of hearing loss. Recent research also found that cochlear macrophages participated in the process of outer hair cell loss in Cx26 mutant mice. The systemic application of dexamethasone can prevent the loss of outer hair cells and improve hearing. The study of Cx26 mutation-related hearing loss should be based on a deeper understanding of the physiological functions and regulatory networks of the Cx26 protein. There is still a long way to go in developing treatments based on the pathological processes of Cx26-related deafness to completely restore hearing.

Cochlear implantation is an effective treatment strategy for patients with hearing loss caused by *GJB2* variants. Gene therapy has also made significant progress in restoring the hearing of patients with hereditary deafness. Adeno-associated virus (AAV)-mediated gene therapy has proved to be helpful in restoring the hearing of *Gjb2* mutant mice. Using an AAV vector, the *Gjb2* gene was successfully expressed in the cochlear Sertoli cells of newborn mice through the round window membrane, leading to significant improvements in the auditory responses and development of the cochlear structure (Yu et al., 2014). With the continuous improvement of AAV serotypes and capsid modifications, this technology will likely contribute to the development of clinical treatments for hereditary deafness.

## Author contributions

YJ and YW wrote the manuscript. WC designed the manuscript. QZ, YX, XG, and SZ provided the reference materials. All authors read and approved the final manuscript.

## References

[B1] Anselmi F. Hernandez V. H. Crispino G. Seydel A. Ortolano S. Roper S. (2008). ATP release through connexin hemichannels and gap junction transfer of second messengers propagate Ca2+ signals across the inner ear. *Proc. Natl. Acad. Sci. U.S.A.* 105 18770–18775. 10.1073/pnas.0800793105 19047635 PMC2596208

[B2] Ashmore J. F. (1987). A fast motile response in guinea-pig outer hair cells: the cellular basis of the cochlear amplifier. *J. Physiol.* 388 323–347. 3656195 10.1113/jphysiol.1987.sp016617 PMC1192551

[B3] Bobbin R. P. Thompson M. H. (1978). Effects of putative transmitters on afferent cochlear transmission. *Ann. Otol. Rhinol. Laryngol.* 87(2 Pt 1), 185–190. 206175 10.1177/000348947808700207

[B4] Brownell W. E. Bader C. R. Bertrand D. Ribaupierre Y d (1985). Evoked mechanical responses of isolated cochlear outer hair cells. *Science* 227 194–196. 3966153 10.1126/science.3966153

[B5] Chai R. Li G. Wang J. Zou J. (2018). hearing loss: reestablish the neural plasticity in regenerated spiral ganglion neurons and sensory hair cells 2018. *Neural Plast.* 2018:4759135. 10.1155/2018/4759135 30647730 PMC6311734

[B6] Chai R. Li H. Yang T. Sun S. Yuan Y. (2022). Editorial: hearing loss: mechanisms and prevention. *Front. Cell Dev. Biol.* 10:838271. 10.3389/fcell.2022.838271 35186939 PMC8850831

[B7] Chen S. Xie L. Xu K. Cao H. Wu X. Xu X. (2018). Developmental abnormalities in supporting cell phalangeal processes and cytoskeleton in the *GJB2*GJB2 knockdown mouse model. *Dis. Model Mech.* 11:dmm033019. 10.1242/dmm.033019 29361521 PMC5894950

[B8] Dallos P. (1992). The active cochlea. *J. Neurosci.* 12 4575–4585. 1464757 10.1523/JNEUROSCI.12-12-04575.1992 PMC6575778

[B9] Dayaratne M. W. Vlajkovic S. Lipski J. Thorne P. R. (2014). Kolliker’s organ and the development of spontaneous activity in the auditory system: implications for hearing dysfunction. *Biomed. Res. Int.* 2014:367939. 10.1155/2014/367939 25210710 PMC4156998

[B10] Defourny J. Peuckert C. Kullander K. Malgrange B. (2019). EphA4-ADAM10 interplay patterns the cochlear sensory epithelium through local disruption of Adherens junctions. *iScience* 11 246–257. 10.1016/j.isci.2018.12.017 30639848 PMC6327856

[B11] Fetoni A. R. Zorzi V. Paciello F. Ziraldo G. Peres C. Raspa M. (2018). Cx26 partial loss causes accelerated presbycusis by redox imbalance and dysregulation of Nfr2 pathway. *Redox Biol.* 19 301–317. 10.1016/j.redox.2018.08.002 30199819 PMC6129666

[B12] Forrest D. Reh T. A. Rusch A. (2002). Neurodevelopmental control by thyroid hormone receptors. *Curr. Opin. Neurobiol.* 12 49–56. 11861164 10.1016/s0959-4388(02)00289-1

[B13] Gabriel H. D. Jung D. Bützler C. Temme A. Traub O. Winterhager E. (1998). Transplacental uptake of glucose is decreased in embryonic lethal connexin26-deficient mice. *J. Cell Biol.* 140 1453–1461. 10.1083/jcb.140.6.1453 9508777 PMC2132681

[B14] Gale J. E. Piazza V. Ciubotaru C. D. Mammano F. (2004). A mechanism for sensing noise damage in the inner ear. *Curr. Biol.* 14 526–529. 15043820 10.1016/j.cub.2004.03.002

[B15] Greenwood D. Jagger D. Huang L. Hoya N. Thorne P. Wildman S. (2007). P2X receptor signaling inhibits BDNF-mediated spiral ganglion neuron development in the neonatal rat cochlea. *Development* 134 1407–1417. 10.1242/dev.002279 17329369

[B16] Griffith A. Yang Y. Pryor S. Park H. Jabs E. Joseph B. Jr. (2006). Cochleosaccular dysplasia associated with a connexin 26 mutation in keratitis-ichthyosis-deafness syndrome. *Laryngoscope* 116 1404–1408. 10.1097/01.mlg.0000224549.75161.ca 16885744 PMC2563154

[B17] Guerra E. Trerotola M. Relli V. Lattanzio R. Tripaldi R. Vacca G. (2021). Trop-2 induces ADAM10-mediated cleavage of E-cadherin and drives EMT-less metastasis in colon cancer. *Neoplasia* 23 898–911. 10.1016/j.neo.2021.07.002 34320447 PMC8334386

[B18] He Z. Guo L. Shu Y. Fang Q. Zhou H. Liu Y. (2017). Autophagy protects auditory hair cells against neomycin-induced damage. *Autophagy* 13 1884–1904. 10.1080/15548627.2017.1359449 28968134 PMC5788479

[B19] He Z. Zou S. Li M. Liao F. Wu X. Sun H. (2020). The nuclear transcription factor FoxG1 affects the sensitivity of mimetic aging hair cells to inflammation by regulating autophagy pathways. *Redox Biol.* 28:101364. 10.1016/j.redox.2019.101364 31731101 PMC6920089

[B20] Housley G. D. Kanjhan R. Raybould N. P. Greenwood D. Salih S. G. Järlebark L. (1999). Expression of the P2X(2) receptor subunit of the ATP-gated ion channel in the cochlea: implications for sound transduction and auditory neurotransmission. *J. Neurosci.* 19 8377–8388. 10.1523/JNEUROSCI.19-19-08377.1999 10493739 PMC6783052

[B21] Huang L. Thorne P. Vlajkovic S. Housley G. (2010). Differential expression of P2Y receptors in the rat cochlea during development. *Purinergic Signal.* 6 231–248. 20806015 10.1007/s11302-010-9191-x PMC2912996

[B22] Inoshita A. Iizuka T. Okamura H. Minekawa A. Kojima K. Furukawa M. (2008). Postnatal development of the organ of Corti in dominant-negative *GJB2*GJB2 transgenic mice. *Neuroscience* 156 1039–1047. 10.1016/j.neuroscience.2008.08.027 18793701

[B23] Jagger D. J. Forge A. (2015). Connexins and gap junctions in the inner ear–it’s not just about K(+) recycling. *Cell Tissue Res.* 360 633–644. 25381570 10.1007/s00441-014-2029-z PMC4452565

[B24] Johnen N. Francart M. Thelen N. Cloes M. Thiry M. (2012). Evidence for a partial epithelial-mesenchymal transition in postnatal stages of rat auditory organ morphogenesis. *Histochem. Cell Biol.* 138 477–488. 10.1007/s00418-012-0969-5 22610129

[B25] Kujawa S. G. Erostegui C. Fallon M. Crist J. Bobbin R. P. (1994). Effects of adenosine 5’-triphosphate and related agonists on cochlear function. *Hear Res.* 76 87–100. 7928720 10.1016/0378-5955(94)90091-4

[B26] Liang C. Zhu Y. Zong L. Lu G. Zhao H. (2012). Cell degeneration is not a primary causer for Connexin26 (*GJB2*) deficiency associated hearing loss. *Neurosci. Lett.* 528 36–41. 22975134 10.1016/j.neulet.2012.08.085 PMC3467974

[B27] Liu W. Xu L. Wang X. Zhang D. Sun G. Wang M. (2021). PRDX1 activates autophagy via the PTEN-AKT signaling pathway to protect against cisplatin-induced spiral ganglion neuron damage. *Autophagy* 17 4159–4181. 10.1080/15548627.2021.1905466 33749526 PMC8726717

[B28] Liu X. Jin Y. Chen S. Xu K. Xie L. Qiu Y. (2021). F-actin dysplasia involved in organ of corti deformity in *GJB2* knockdown mouse model. *Front. Mol. Neurosci.* 14:808553. 10.3389/fnmol.2021.808553 35345836 PMC8957075

[B29] Majumder P. Crispino G. Rodriguez L. Ciubotaru C. Anselmi F. Piazza V. (2010). ATP-mediated cell-cell signaling in the organ of Corti: the role of connexin channels. *Purinergic Signal* 6 167–187. 10.1007/s11302-010-9192-9 20806010 PMC2912995

[B30] Maretzky T. Reiss K. Ludwig A. Buchholz J. Scholz F. Proksch E. (2005). ADAM10 mediates E-cadherin shedding and regulates epithelial cell-cell adhesion, migration, and beta-catenin translocation. *Proc. Natl. Acad. Sci. U.S.A.* 102 9182–9187. 10.1073/pnas.0500918102 15958533 PMC1166595

[B31] Maretzky T. Scholz F. Köten B. Proksch E. Saftig P. Reiss K. (2008). ADAM10-mediated E-cadherin release is regulated by proinflammatory cytokines and modulates keratinocyte cohesion in eczematous dermatitis. *J. Invest. Dermatol.* 128 1737–1746. 10.1038/sj.jid.5701242 18200054

[B32] Minekawa A. Abe T. Inoshita A. Iizuka T. Kakehata S. Narui Y. (2009). Cochlear outer hair cells in a dominant-negative connexin26 mutant mouse preserve non-linear capacitance in spite of impaired distortion product otoacoustic emission. *Neuroscience* 164 1312–1319. 10.1016/j.neuroscience.2009.08.043 19712724

[B33] Muñoz D. J. Thorne P. R. Housley G. D. Billett T. E. Battersby J. M. (1995). Extracellular adenosine 5’-triphosphate (ATP) in the endolymphatic compartment influences cochlear function. *Hear Res.* 90 106–118. 8974987 10.1016/0378-5955(95)00152-3

[B34] Nikolic P. Housley G. Thorne P. (2003). Expression of the P2X7 receptor subunit of the adenosine 5’-triphosphate-gated ion channel in the developing and adult rat cochlea. *Audiol. Neurootol.* 8 28–37. 10.1159/000067891 12566690

[B35] Nin F. Hibino H. Murakami S. Suzuki T. Hisa Y. Kurachi Y. (2012). Computational model of a circulation current that controls electrochemical properties in the mammalian cochlea. *Proc. Natl. Acad. Sci. U.S.A.* 109 9191–9196. 10.1073/pnas.1120067109 22619324 PMC3384130

[B36] Piazza V. Ciubotaru C. D. Gale J. Mammano F. (2007). Purinergic signalling and intercellular Ca2+ wave propagation in the organ of Corti. *Cell Calcium* 41 77–86. 16828497 10.1016/j.ceca.2006.05.005

[B37] Qiu Y. Xu K. Xie L. Chen S. Sun Y. (2022). The reduction in microtubule arrays caused by the dysplasia of the non-centrosomal microtubule-organizing center leads to a malformed organ of Corti in the Cx26-null mouse. *Biomedicines* 10:1364. 10.3390/biomedicines10061364 35740388 PMC9219875

[B38] Reyat J. S. Chimen M. Noy P. Szyroka J. Rainger G. Tomlinson M. (2017). ADAM10-interacting tetraspanins Tspan5 and Tspan17 regulate VE-cadherin expression and promote T lymphocyte transmigration. *J. Immunol.* 199 666–676. 10.4049/jimmunol.1600713 28600292 PMC5502317

[B39] Santos-Sacchi J. Dallos P. (1983). Intercellular communication in the supporting cells of the organ of Corti. *Hear Res.* 9 317–326. 6841286 10.1016/0378-5955(83)90034-5

[B40] Seipold L. Altmeppen H. Koudelka T. Tholey A. Kasparek P. Sedlacek R. (2018). *In vivo* regulation of the A disintegrin and metalloproteinase 10 (ADAM10) by the tetraspanin 15. *Cell Mol. Life Sci.* 75 3251–3267. 10.1007/s00018-018-2791-2 29520422 PMC11105247

[B41] Spray D. C. Ye Z. C. Ransom B. R. (2006). Functional connexin “hemichannels”: a critical appraisal. *Glia* 54 758–773. 10.1002/glia.20429 17006904

[B42] Stout C. Costantin J. Naus C. Charles A. C. (2002). Intercellular calcium signaling in astrocytes via ATP release through connexin hemichannels. *J. Biol. Chem.* 277 10482–10488. 11790776 10.1074/jbc.M109902200

[B43] Teubner B. Michel V. Pesch J. Lautermann J. Cohen-Salmon M. Söhl G. (2003). Connexin30 (Gjb6)-deficiency causes severe hearing impairment and lack of endocochlear potential. *Hum. Mol. Genet.* 12 13–21. 10.1093/hmg/ddg001 12490528

[B44] Tritsch N. X. Rodríguez-Contreras A. Crins T. H. Wang H. Borst J. G. Bergles D. E. (2010). Calcium action potentials in hair cells pattern auditory neuron activity before hearing onset. *Nat. Neurosci.* 13 1050–1052. 20676105 10.1038/nn.2604 PMC2928883

[B45] Tritsch N. X. Yi E. Gale J. E. Glowatzki E. Bergles D. E. (2007). The origin of spontaneous activity in the developing auditory system. *Nature* 450 50–55. 17972875 10.1038/nature06233

[B46] Wang M. Dong Y. Gao S. Zhong Z. Cheng C. Qiang R. (2022). Hippo/YAP signaling pathway protects against neomycin-induced hair cell damage in the mouse cochlea. *Cell Mol. Life Sci.* 79:79. 10.1007/s00018-021-04029-9 35044530 PMC8770373

[B47] Whitlon D. S. (1993). E-cadherin in the mature and developing organ of Corti of the mouse. *J. Neurocytol.* 22 1030–1038. 8106878 10.1007/BF01235747

[B48] Yu Q. Wang Y. Chang Q. Wang J. Gong S. Li H. (2014). Virally expressed connexin26 restores gap junction function in the cochlea of conditional *GJB2*GJB2 knockout mice. *Gene Ther.* 21 71–80. 10.1038/gt.2013.59 24225640 PMC3881370

[B49] Zhang H. Xie L. Chen S. Qiu Y. Sun Y. Kong W. (2022). Thyroxine regulates the opening of the organ of corti through affecting P-cadherin and acetylated microtubule. *Int. J. Mol. Sci.* 23:13339. 10.3390/ijms232113339 36362134 PMC9656988

[B50] Zhang Y. Fang Q. Wang H. Qi J. Sun S. Liao M. (2023). Increased mitophagy protects cochlear hair cells from aminoglycoside-induced damage. *Autophagy* 19 75–91. 35471096 10.1080/15548627.2022.2062872 PMC9809934

[B51] Zhang Y. Tang W. Ahmad S. Sipp J. Chen P. Lin X. (2005). Gap junction-mediated intercellular biochemical coupling in cochlear supporting cells is required for normal cochlear functions. *Proc. Natl. Acad. Sci. U.S.A.* 102 15201–15206. 10.1073/pnas.0501859102 16217030 PMC1257692

[B52] Zhao H. B. (2017). Hypothesis of K(+)-recycling defect is not a primary deafness mechanism for Cx26 (GJB2) deficiency. *Front. Mol. Neurosci.* 10:162. 10.3389/fnmol.2017.00162 PMC5445178 28603488

[B53] Zhao H. B. Santos-Sacchi J. (1999). Auditory collusion and a coupled couple of outer hair cells. *Nature* 399 359–362. 10.1038/20686 10360573

[B54] Zhong Z. Fu X. Li H. Chen J. Wang M. Gao S. (2020). Citicoline protects auditory hair cells against neomycin-induced damage. *Front. Cell Dev. Biol.* 8:712. 10.3389/fcell.2020.00712 32984303 PMC7487320

